# Perils and payoffs for patients in serious illness conversations as described by physicians: a qualitative study

**DOI:** 10.1136/bmjoq-2023-002734

**Published:** 2024-05-23

**Authors:** Rebecca Baxter, Susanna Pusa, Sofia Andersson, Anna Sandgren

**Affiliations:** 1 Department of Health and Caring Sciences, Linneaus University, Växjö, Sweden; 2 Department of Nursing, Umeå University, Umeå, Sweden

**Keywords:** Patient-centred care, Communication, Healthcare quality improvement, Hospital medicine, Palliative Care

## Abstract

**Background:**

The Serious Illness Care Programme was developed to promote more, better and earlier serious illness conversations. Conversations about goals and values are associated with improved experiences and outcomes for seriously ill patients. Clinicians’ attitudes and beliefs are thought to influence the uptake and performance of serious illness conversations, yet little is known about how clinicians perceive the impact of these conversations on patients. This study aimed to explore physicians’ perceptions regarding the impact of serious illness conversations for patients.

**Methods:**

The Serious Illness Care Programme was implemented as a quality improvement project in two hospitals in Southern Sweden. Focus group evaluation discussions were conducted with 14 physicians and inductive thematic analysis was undertaken.

**Results:**

The results revealed that physicians considered potential perils and optimised potential payoffs for patients when engaging in serious illness conversations. Potential perils encompassed inappropriate timing, damaging emotions and shattering hopes. Potential payoffs included reflection time, secure space, and united understandings.

**Conclusions:**

Physicians depicted a balance in evaluating the perils and payoffs of serious illness conversations for patients and recognised the interrelation of these possibilities through continual assessment and adjustment.

**WHAT IS ALREADY KNOWN ON THIS TOPIC**

- Serious illness conversations are associated with improved experiences and outcomes for seriously ill patients and their clinicians. How clinicians perceive these conversations can influence the ways in which they are implemented in clinical practice.

**WHAT THIS STUDY ADDS**

- Physicians described consideration of potential perils and optimisation of potential payoffs for patients when considering the impact of serious illness conversations.

**HOW THIS STUDY MIGHT AFFECT RESEARCH, PRACTICE OR POLICY**

- The findings can be used to inform serious illness conversation training and clinical practice initiatives, and highlights the importance of undertaking research to explore clinicians’ attitudes, values and beliefs in relation to implementing communication interventions.

## Introduction

The Serious Illness Care Programme (SICP) is a care delivery model that aims to promote more, better and earlier conversations between seriously ill patients and clinicians.^1 2^ Serious illness conversations endeavour to discover what is most important to patients so that their individual wants and needs can be reflected in their care.^3^ The SICP is informed by the principles of goal-concordant person-centred care and shared decision-making.^4^ Implementation of the SICP has been found to result in decreased patient anxiety and depression,^5^ improved experiences for patients and clinicians^6 7^ and reduced total medical costs.^8^

Effective communication is recognised as a cornerstone of person-centred care.^9^ Studies indicate that patient-centred communication interventions can result in improved physician–patient relationships and patient outcomes.^10 11^ Likewise, poor communication is associated with negative patient experiences and healthcare outcomes.^12 13^ By centring conversations around patients’ wants and needs, physicians are better able to facilitate care that is concordant with the patient’s goals, values, priorities and preferences.^6^

Physician attitudes towards patient-centred communication have been associated with greater intention to involve patients in medical decision-making.^14^ A positive attitude has also been noted in several studies as a significant predictor of behaviour intention in shared decision-making processes between physicians and patients.^15 16^ A study describing organisational factors related to implementing the SICP found that clinicians’ confidence, motivation and attitudes to enacting serious illness conversations could act as barriers as well as facilitators to the implementation.^17^ Likewise, physicians’ attitudes towards talking about death and their perception of whether it was the ‘right’ time to talk about certain issues have been found to impact the ways in which patients were identified for serious illness conversations.^18^ It is therefore important to examine clinician perceptions surrounding communication initiatives, as personal or professional outlooks can impact the success of implementations in practice. This study aimed to explore physicians’ perceptions regarding the impact of serious illness conversations on patients.

## Materials and methods

### Study design

This qualitative study has been conducted and reported in accordance with the consolidated criteria for reporting qualitative research (COREQ).^19^ Reflective thematic analysis was undertaken using an inductive approach as outlined by Braun and Clarke to generate insights and interpretations about the phenomena of interest based on participant experiences.^20^

### Context

The SICP is a comprehensive and structured intervention founded on evidence-based communication strategies to augment dialogue with seriously ill patients.^1^ The programme was developed by Ariadne Labs (Boston, USA)^4^ and has been adapted for the Swedish healthcare setting.^21^ The SICP is comprised of implementations at organisation, management, clinician, family and patient levels. Healthcare providers are supported to implement the SICP through the provision of tools, training and structured support.^1^ The SICP was implemented as a quality improvement project in two hospitals in Southern Sweden. The project was implemented in 20 hospital units in 2017 and 2018. Physicians (n=106) participated in eight hours of serious illness conversation training that included lectures and practice sessions designed to build the necessary knowledge and skills to undertake serious illness conversations.

### Participants and setting

Hospital leaders granted permission for the SICP to be implemented as part of a quality improvement project and for the participating physicians to take part in focus group discussions as part of the project evaluation. Participants were eligible for inclusion if they (1) were qualified physicians and (2) participated in the SICP implementation. Participants were provided with information about the focus group discussions and verbal informed consent was obtained. Department managers invited physicians who participated in the SICP implementation to take part in focus group evaluation discussions. Because participation was voluntary and open to all physicians who engaged in the SICP implementation at the time of the evaluation, invitation and non-participation rates and characteristics were not explored. Fourteen physicians took part in four focus group discussions (table 1). In total, nine men and five women participated with an age range of 39–61 years (mean, 51 years). All participating physicians were hospitalists working in cardiology, endocrinology, haematology or palliative care.

**Table 1:** Focus group discussion participants (N=14)

Focus group	Participants	Duration
Focus group 1	n=5	61 min
Focus group 2	n=4	37 min
Focus group 3	n=3	41 min
Focus group 4	n=2	38 min

### Data collection

Four focus group discussions took place between September 2017 and February 2018 at the participants’ workplace. The semi-structured discussions comprised of questions concerning serious illness conversation training, the Serious Illness Conversation Guide, how patients were identified for conversations and how serious illness conversations were conducted. The questions were formed as open-ended queries to allow participants to discuss the aspects they considered relevant. Only data relevant to the study aim were analysed. Pusa *et al* 22 reported on other sections of the data, focusing on preparedness, impact and compilation of the overall SICP implementation. Two female researchers who were not part of the author group moderated the focus group discussions as moderators. One moderator had a background in nursing and the other in physiotherapy. Both had experience in conducting focus group discussions and had no established relationships with the participants prior to the focus group discussions. The discussions ranged in length from 37 to 61 min with an average duration of 44 min. All discussions were audio-recorded and transcribed for analysis.

### Data analysis

Data were analysed using Braun and Clark’s^20^ six-phase thematic analysis framework: (1) familiarising with the data, (2) generating initial codes, (3) searching for themes, (4) reviewing the themes, (5) defining and naming and (6) producing the report. To obtain a general impression of the data the first author (RB) read the transcribed discussions to search for overall patterns, meanings and initial ideas for coding. The second (SP) and last authors (AS) read the transcripts independently. Transcripts were examined line-by-line and colours were assigned to possible codes. The first author (RB) analysed the list of codes and organised them into groups. This iterative process involved a back-and-forth movement from individual parts of the text to the text as a whole. Thereafter, initial themes and subthemes were generated and revised within the author group until all agreed that the themes, subthemes and supporting data aligned in a meaningful way. The wording of the themes and subthemes were refined and their content expounded. Supporting quotations are provided in italics and denoted by focus group number.

### Translation procedure

The focus group discussions were held and transcribed in Swedish. The initial data analysis was undertaken in the original language to ensure that the meanings and patterns accurately reflected participants’ perceptions in their own words. Once the patterns, meanings and codes were decided, the authors translated the quotes into English for further development into themes and subthemes. The supporting quotations were modified to achieve equivalence in understanding and meaning.

## Results

Physicians perceived the impact of serious illness conversations for patients as encompassing consideration of potential perils and optimisation of potential payoffs (table 2). Considering the perils involved: inappropriate timing, damaging emotions and shattering hopes. Optimising payoffs comprised: reflection time, secure space and united understandings. The two main themes were interdependent and reflected a sense of symbiosis, as the potential for both perils and payoffs influenced how the conversation was perceived to impact patients. This involved weighing the likelihood of negative consequences or reactions with the prospect of positive or beneficial outcomes. The results denote the different yet connected aspects in the perpetual (re)assessment of the existent line between perceived perils and payoffs for patients within the context of serious illness conversations.

**Table 2:** Perceived impact of serious illness conversations for patients

Themes	Subthemes
Considering potential perils	Inappropriate timing Damaging emotions Shattering hopes
Optimising potential payoffs	Reflection time Secure space United understandings

### Considering potential perils

Physicians expressed concern that serious illness conversations could present perils or pitfalls for patients that required careful and constant consideration both in and beyond the conversation interaction.

#### Inappropriate timing

Physicians considered patient symptoms, stability and readiness in relation to conversation timing. It was important not to engage in a conversation too early or too late, as the act of having a serious illness conversation was thought to prompt patients to consider their future in different ways. If conducted too early, physicians felt that patients could suffer psychological or spiritual harm. If conducted too late, physicians believed that patients could be damaged through lack of knowledge or preparation, and that they would not experience the benefits of early intervention or care integration.

… that you find the right patient in good time and that, not the week before they die. (Focus group 3)

Inappropriate timing extended beyond the illness trajectory to the patient’s immediate reality. It was perceived to be difficult for patients to have a conversation about serious illness if they were not relieved of physical symptoms such as shortness of breath, pain or distress; however, physicians also recognised that these symptoms were common for many seriously ill patients which could make it difficult to reconcile conversations in practice.

…if the patient is always depressed or anxious or worried or something else, or has pain or something, and then it is very difficult to find the moment when we can talk to that patient. (Focus group 1)

#### Damaging emotions

Physicians were conscious that talking about serious illness could evoke feelings of discomfort, sadness, anxiety or worry in patients. Physicians perceived that for some patients the conversation could lead to increased anxiety by drawing attention to the likelihood of becoming more unwell in the future.

Yes, because it drags on, [the patient thinks] ‘yes, but when I'm fine now, why should I have to think about getting sick again?’ It creates a lot of anxiety and worry and may not lead to anything good. (Focus group 2)

They acknowledged that the conversation could cause patients to worry about their situation, especially when discussing the topic of prognosis. In this way, serious illness conversations were perceived as having the potential to make the patient’s emotional state worse by introducing imposing or intrusive thoughts.

… it just brings up a lot of worry and anxiety and … maybe the rest of their lives will be hell. And you don’t want to inflict that on them*…*. (Focus group 2)

#### Shattering hopes

Physicians felt that focusing on problems and difficulties during serious illness conversations had the potential to shatter hope and the will to live. There was a perception that concentrating on discussing a patient’s illness could cause them to ruminate and possibly lead to an existential crisis. Serious illness conversations could leave patients feeling agitated or dejected which was sensed by physicians to add to their overall illness burden. Shattered hopes extended to patient relatives and their loved ones as the conversation was seldom perceived to impact one person alone.

And if it only concerns the patient, maybe it’s easier, but if it concerns relatives and loved ones sitting next to them who have had a great life… you [the physician] come and say, ‘no, but your love is over now’. (Focus group 3)

Physicians were conscious that patients could be harmed by having false hope, but faced a different kind of harm if hope was taken away without warning. Patients who were not aware of, or did not accept, the reality of their prognosis were thought to be at greater risk of losing hope during serious illness conversations.

It’s difficult. And then we take away from the patient something that is also important, which is hope. To be able to live on, you have to believe for quite a long time that there is hope. (Focus group 1)

### Optimising potential payoffs

Serious illness conversations were recognised as encompassing potential payoffs and benefits for patients that physicians attempted to maximise throughout the discussion.

#### Reflection time

The conversation was viewed as an opportunity for patients to slow down, breathe and have time to reflect. Physicians felt that healthcare processes could feel stressful and overwhelming for patients. Serious illness conversations were perceived to provide patients with the chance to discuss and calmly reflect on their options. There was a perception that serious illness conversations were an investment for patients that would pay off in the future.

…difficult conversations now facilitate difficult decisions later. (Focus group 1)

Physicians spoke about serious illness conversations as an opportunity for patients to think about what they wanted. Time to reflect enabled patient preparation for future decisions and conversations without the added distraction or pressure that can occur when combining communication with other care tasks.

…it shouldn’t be a breaking point situation, but it should be a situation where you think that right now it’s a bit calm and time for some kind of breathing or reflection or something. (Focus group 1)

#### Secure space

The conversation was perceived to offer patients a secure and safe space to discuss their feelings where they could feel seen and heard within and beyond their illness. Physicians perceived the conversation as being a place for patients to express themselves, both verbally and emotionally. This was viewed by physicians as beneficial for patients because it supported the development of a holistic understanding of their situation and needs.

I think it is above all about how to deal with emotions and also to ask questions that actually evoke emotions. Yes, and to sort of dare to go a little deeper, to not only go into the purely medical… here you sort of go a little deeper and dig down on what can be a bit troublesome. (Focus group 3)

Physicians reflected that supporting patients to express themselves presented possibilities to process complex sentiments, which could help patients to better understand, acknowledge or accept their situation. Consciously giving patients space to express themselves was perceived to facilitate communication that was appropriate and sensitive.

I can create a feeling in the patient that here is someone who has listened to me. (Focus group 1)

#### United understandings

United understandings were described by physicians as comprising mutual acknowledgement of patient desires and inclusion in deciding what to do next. Physicians perceived that serious illness conversations supported development of a common relational ground where patients could identify and share their wants and needs.

… make sure to have the conversation early so that you agree with the patient on how to plan and how to prioritize. (Focus group 2)

Expression of these desires was viewed by physicians as being facilitated through a comfortable partnership and shared understandings. Serious illness conversations were depicted by physicians as a convergence that adapted to different patient needs through the provision of information and reassurance. Establishing a sense of togetherness was thought to enhance the relationship between the physician and the patient by building mutual trust and respect during the encounter.

They [the patient] knew that ‘if I get much worse, I want to be cared for at home as much as possible’…I think they appreciated that I had questions about how they wanted it. (Focus group 2)

## Discussion

This study explored physicians’ perceptions regarding the impact of serious illness conversations. The results highlight the existent duality in assessing and balancing perils and payoffs when undertaking serious illness conversations. Physicians sought to avoid pitfalls and maximise positive experiences where possible, but they were also aware that outcomes could change over time and with perspective. The results highlight that subjectivity and temporality exists within physicians’ perceptions of clinical communication, and that the approach and appropriateness of serious illness conversations for patients must be continually evaluated.

Perceptions surrounding the impact of serious illness conversations, whether characterised as positive or negative, can play a crucial role in driving systemic and organisational improvements.^23^ The findings in this study revealed concerns related to timing, emotional distress and diminishing hope. The temporal dimension within serious illness conversations, and the challenges associated with timing, have been described in prior research.^23 24^ The process of identifying the right patient at the right time involves both ethical and existential aspects.^18^ Concern about eroding patient hope is intertwined with the challenge of appropriate timing of conversations^18^ and is situated within the complex nature of clinician-patient interactions.^25^ The findings from the present study support that of another patient-centred communication intervention that also noted associations between discussion domains and clinician perceptions.^10^ For example, the provision of emotional support might depend on the clinician’s subjective discernment of the patient’s responses during different parts of the conversation. Taken together, elucidating both patient and clinician perspectives may facilitate reflective and careful conversations that meet the needs of all who are involved in sensitive communication.

Cultivating rapport between clinicians, patients and relatives can lead to more proactive roles in care-related conversations. This approach encourages an open dialogue and facilitates coherent discussions on existential matters.^26^ Clinicians’ perception of established trust can serve as a guiding factor in navigating sensitive disclosures in serious illness conversations.^27^ Furthermore, serious illness conversations can fortify therapeutic relationships by providing emotional support, fostering partnership in treatment decisions and strengthening overall relationships. This, in turn, could enhance patient and family satisfaction with decisions and instil greater assurance among clinicians that the care being provided aligns with patients’ expressed wishes.^23^ The significance of relationship building is underscored by the findings of this study, wherein serious illness conversations were perceived to provide patients with an environment that could promote shared relational ground.

The findings of the present study suggest that physicians are cognisant that discussing serious illness could evoke feelings of distress and intense emotions in patients. Previous studies emphasise the significance of emotion-handling skills in praxis.^4 28 29^ Discomfort in handling emotions can therefore function as an inhibitor for serious illness conversations.^23^ Discomfort and uncertainty may manifest in the presence of strong emotions, particularly when concerns arise about potentially upsetting the patient.^30^ In the context of serious illness conversations, Wasp *et al* 27 explored emotion regulation concerning how clinicians handle both their own and their patients’ emotions. The findings indicate that clinicians may have incomplete awareness of their emotion-handling and regulation strategies.^27^ Culture-related factors, including clinician beliefs, attitudes and behaviours, can likewise affect engagement in serious illness conversations, serving as either enablers or barriers.^23^ Furthermore, clinician mindset and attitudes can impact proficiency to engage in serious illness conversations. Targeted learning objectives related to clinician attitudes could emphasise the identification of personal barriers hindering engagement in such conversations. Training methods aligned with this goal may incorporate exercises to enhance clinician comfort, practical demonstrations and debriefing regarding the use of the Serious Illness Conversation Guide, as well as highlighting evidence demonstrating the positive impact of serious illness conversations on patient outcomes.^31^ Educational interventions with this focus have led to improvements in clinical healthcare settings.^31^ Communication training and support should focus on creating a space for clinicians to enhance their awareness of, and ability to reflect on, their own guiding attitudes, values and beliefs.^32^

### Strengths and limitations

This study takes an educational approach by addressing perceived challenges and benefits for patients from the physician’s perspective regarding serious illness conversations. The findings could inform learning perspectives when implementing the SICP by elucidating clinicians’ beliefs regarding serious illness conversations early in the implementation process in order to adapt and tailor the conversation to both the clinician and the patient. As transferability is difficult to assess, training initiatives should be adapted to the specific setting guided by identification and awareness of clinicians’ beliefs and perceptions regarding the impact of serious illness conversations for patients. Purposive sampling methods allowed for the collection of data from a targeted group of participants who were able and willing to discuss the subject of interest; however, this form of sampling can result in selection bias as the research aim and participant motivations can impact the study.^33^ To mitigate this risk, a full description of the researchers, setting and participants has been provided to ensure that the findings are not extrapolated beyond their scope. The participants were limited to physicians, but other professions have been involved in other SICP implementations and their perspectives warrant exploration in future studies. Lastly, this study elucidates the perceived perils and payoffs for patients from the perspective of physicians; however, it is important to note that this may not necessarily align with the experiences of seriously ill patients or their family members. It would be useful to explore and compare patient and clinician perceptions in future research.

## Conclusions

This study revealed that physicians perceived and balanced perils and payoffs for patients when considering the impact of serious illness conversations. Physicians’ own understandings of the impact of the conversation influenced the perceived value of the conversation for patients. These findings contribute to informing future education and interventions aimed at improving serious illness communication between physicians and patients.

## Data Availability

No data are available. The data are not publicly available.
